# Factors associated with life satisfaction of Korean older adults with disabilities living alone: a cross-sectional study using the ICF framework

**DOI:** 10.3389/fpubh.2026.1812101

**Published:** 2026-06-10

**Authors:** Song Yi Han, Young Ko, Hye-Young Jang

**Affiliations:** 1 Department of Nursing Science, Sunmoon University, Asan-si, Republic of Korea; 2 College of Nursing, Gachon University, Incheon, Republic of Korea; 3 College of Nursing, Hanyang University, Seoul, Republic of Korea

**Keywords:** environmental factor, ICF model, life satisfaction, living alone, older adults with disabilities

## Abstract

**Background/objectives:**

This study aims to identify factors associated with life satisfaction among older adults with disabilities living alone using an analytic framework informed by the International Classification of Functioning, Disability and Health (ICF).

**Methods:**

Data were analyzed from 536 older adults (aged 60 years and older) with disabilities using the fourth wave of the Disability and Life Dynamics Panel conducted by the Korea Disability Development Institute. Factors associated with life satisfaction were categorized into personal factors (sex, education level, employment status, subjective economic hardship, self-esteem, and disability acceptance), environmental factors (number of close persons and frequency of contact with them), health-related characteristics (subjective health status, primary disability type, and disability severity), and activities and participation (needs for assistance with activities of daily living, social activity participation, online social interaction, and leisure activity participation). Statistical analyses included descriptive statistics, *t*-tests, analysis of variance, Pearson’s correlation coefficients, and hierarchical regression analysis.

**Results:**

Subjective economic hardship was negatively associated with life satisfaction (*β* = −0.151, *p* < 0.001), whereas employment status (*β* = 0.326, *p* < 0.001), self-esteem (*β* = 0.114, *p* = 0.002), and disability acceptance (*β* = 0.101, *p* = 0.006) were positively associated with life satisfaction within the personal factors domain. Among environmental factors, rare contact with close persons was negatively associated with life satisfaction (*β* = −0.121, *p* = 0.001). Regarding health-related characteristics, subjective health status was positively associated with life satisfaction (*β* = 0.256, *p* < 0.001). For activities and participation, unmet needs for assistance with activities of daily living (*β* = −0.078, *p* = 0.013) and non-participation in leisure activities (*β* = −0.073, *p* = 0.016) were negatively associated with life satisfaction.

**Conclusion:**

This study highlights the multidimensional nature of life satisfaction among older adults with disabilities living alone, demonstrating that both individual and environmental factors are significantly associated with life satisfaction. Future initiatives should focus on expanding job programs, promoting social and leisure activities, and developing integrated health and social support interventions that consider both individual and community contexts, along with improving age-friendly environments.

## 1 Introduction

With the rapid progression of population aging, the number of older adults is increasing substantially, accompanied by a concomitant rise in the population of older adults with disabilities (1, 2). Older adults with disabilities are susceptible to complex and overlapping challenges arising not only from the physiological changes associated with normal aging but also from the physical and social limitations imposed by their disabilities (3). In South Korea, the proportion of adults aged 65 years and older among the total population of persons with disabilities increased from 49.9% in 2020 to 54.3% in 2023, indicating a pronounced trend toward population aging within the disability population (2). In addition, as household structures continue to change and the number of co-residing family members declines, the proportion of older adults with disabilities living alone has remained consistently high and continues to increase (2). These individuals are particularly vulnerable to a range of issues, including diminished physical function, social isolation, restricted participation in leisure activities, and economic hardship (4, 5). Such compounded vulnerabilities not only adversely affect individual quality of life but also present significant public health challenges related to social care and health management.

Life satisfaction is widely conceptualized as the cognitive component of happiness or subjective well-being, reflecting individuals’ evaluative judgments about their life circumstances. According to Diener (6), life satisfaction represents a cognitive appraisal of one’s life based on personal standards and expectations, emphasizing individuals’ perceptions, evaluations, and judgments of their life situations rather than emotional states. Campbell et al. (7) further suggested that life satisfaction can be assessed not only as overall satisfaction with life but also as satisfaction across specific life domains, making it a particularly useful and reliable construct for research on subjective quality of life. This domain-based approach allows for more concrete measurement and facilitates the identification of areas in which interventions and policies may improve individuals’ quality of life. Consistent with this conceptualization, the Disability and Life Dynamics Panel (DLDP) operationalizes life satisfaction as a composite indicator based on subjective evaluations across multiple life domains, and the present study follows this operational definition.

This conceptualization of life satisfaction is particularly relevant for populations experiencing cumulative and intersecting disadvantages, for whom subjective evaluations of life conditions may be shaped by multiple constraints and stressors. Life satisfaction therefore serves as an important psychological indicator reflecting how positively individuals evaluate their overall lives and has increasingly been used as a key measure of quality of life (8). Previous studies have reported that life satisfaction among older adults and people with disabilities is influenced by a diverse range of factors. Economic stability and employment have been identified as important factors associated with subjective well-being, whereas economic hardship has consistently been associated with lower life satisfaction (9, 10). Psychological resources, specifically high self-esteem and disability acceptance, may help individuals better adapt to the negative effects of physical impairments and disability-related challenges (11, 12).

Furthermore, social connectedness—including social participation and frequent contact with close persons—has been recognized as an important factor in reducing social isolation and promoting social connectedness in this population (13–16). Health-related variables, such as subjective health status and functional independence in daily living, have also been identified as important correlates of life satisfaction (17–19). Despite this extensive body of research, most previous studies have focused on either older adults in general (10, 13) or the broader disability population (14, 20, 21). Relatively few studies have specifically examined older adults with disabilities living alone, a population experiencing multiple and intersecting vulnerabilities (20, 22).

The International Classification of Functioning, Disability and Health (ICF) model, proposed by the World Health Organization (WHO), provides a conceptual framework for understanding health and disability as multidimensional phenomena encompassing body functions and structures, activities, participation, personal factors, and environmental contexts (23, 24). The ICF model conceptualizes disability not solely as a physical impairment but as an outcome of interactions between individual characteristics and social and environmental conditions (23, 24). An integrated approach informed by the ICF framework is therefore useful for comprehensively examining factors associated with life satisfaction in vulnerable populations.

To address this gap, this study applies an ICF-informed analytic framework to examine whether previously identified determinants remain significantly associated with life satisfaction and how multidimensional factors collectively relate to life satisfaction within the context of living alone. Based on the ICF-informed analytic framework and prior empirical findings, we formulated the following hypotheses:

H1: Personal factors, including economic conditions and psychological resources, would be significantly associated with life satisfaction.

H2: Environmental factors related to social connectedness would be significantly associated with life satisfaction.

H3: Health-related factors would be significantly associated with life satisfaction.

H4: Activities and participation factors would be significantly associated with life satisfaction.

The findings of this study may provide foundational evidence for the development of context-sensitive policies and programs aimed at improving quality of life and social integration among older adults with disabilities.

## 2 Materials and methods

### 2.1 Study design and participants

This study employed a descriptive secondary data analysis using data from the 4th wave of the Disability and Life Dynamics Panel (DLDP). The study was guided by the ICF framework developed by the World Health Organization (25) and aimed to identify factors associated with life satisfaction among older adults with disabilities living alone.

### 2.2 Data source and ethical considerations

Data were obtained from the fourth wave of the Disability and Life Dynamics Panel (DLDP), a nationally representative longitudinal survey conducted by the Korea Disability Development Institute (KODDI). The DLDP was designed to examine the demographic characteristics, health status, social participation, independence, and living conditions of persons with disabilities in South Korea over time. Access to the dataset was granted after formal application and approval through the Disability Statistics Data Portal (https://koddi.or.kr/stat/html/user/main/main).

The panel survey was constructed using individuals who were officially registered as persons with disabilities with the Ministry of Health and Welfare between January 1, 2015, and December 31, 2017, excluding those residing in institutional care facilities. The survey was conducted using structured face-to-face interviews administered by trained professional interviewers, following standardized survey protocols to ensure consistency and data quality. The panel was established in 2018 with 6,121 participants, and the baseline survey (Wave 1) was carried out in the same year. The DLDP is conducted on an annual basis, and the 4th wave of the survey, which was used in the present study, was based on this nationally representative panel.

For the present study, individuals aged 60 years or older who were living alone were selected. After excluding cases with missing data, 536 participants were included in the final analysis.

The study was conducted with the approval from the Institutional Review Board (IRB) of author’s institution (IRB No. SM-202512-030-1).

### 2.3 Measure

**Figure 1:**
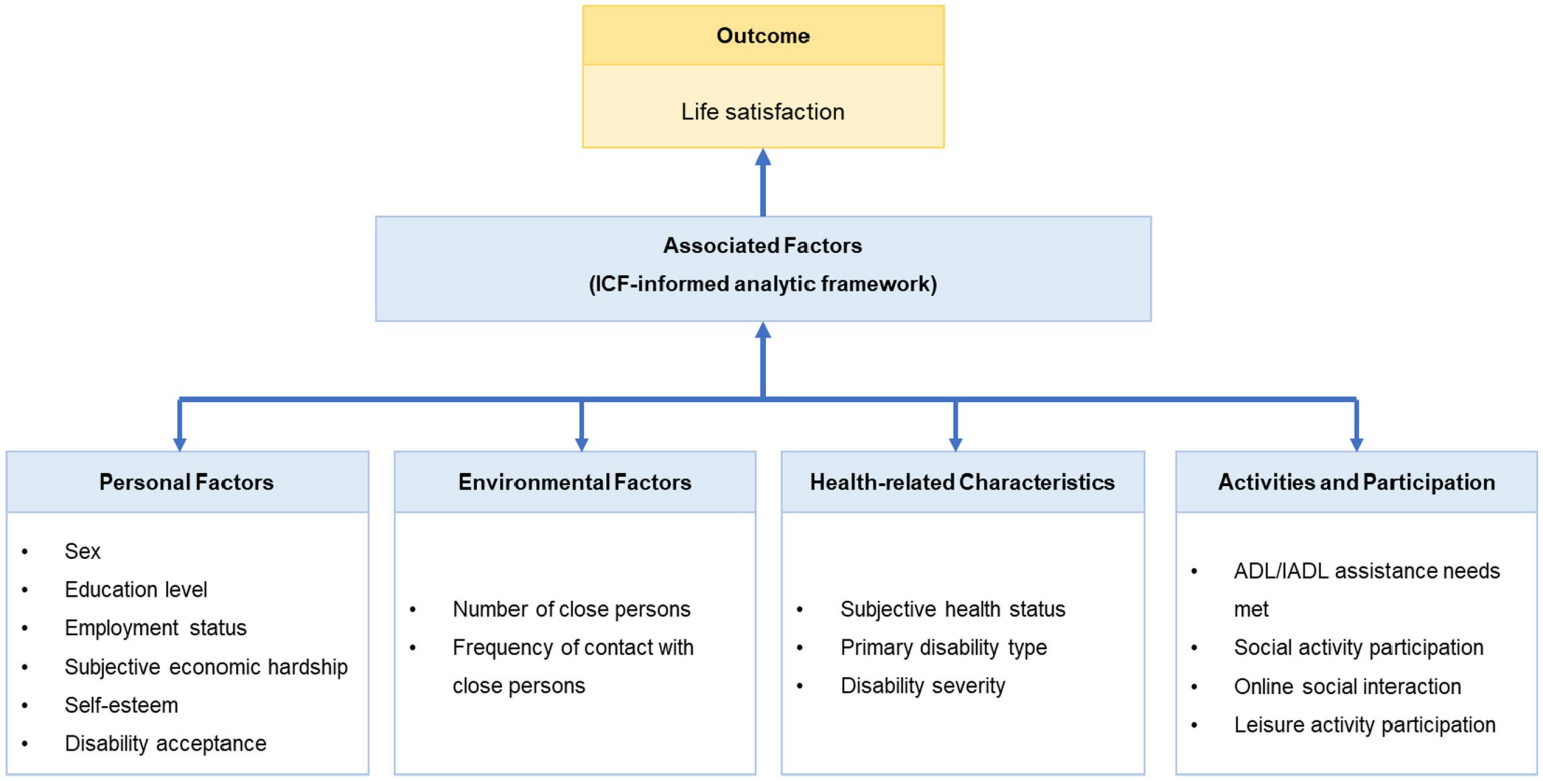
Analytic framework of the study informed by the ICF.

Study variables were organized using an ICF-informed analytic framework, encompassing personal factors, environmental factors, health-related characteristics, and activities and participation (Figure 1).

#### 2.3.1 Personal factors

In this study, personal factors were defined, in accordance with the ICF framework, as individual background characteristics and psychological resources that are not classified as health conditions but may influence how individuals experience disability and daily life. Accordingly, personal factors included sex, education level, employment status, subjective economic hardship, self-esteem and disability acceptance.

Sex was categorized as male or female. Education level was categorized as no formal education, elementary school, middle school, high school, and college or higher. Employment status was categorized as employed or unemployed. Subjective economic hardship was assessed using an 8-item scale measuring perceived difficulties related to basic living expenses (food, clothing, and housing) (26). Each item was rated on a 4-point rating scale (1 = not at all, 4 = always). An example item is “I find it difficult to pay monthly utility bills.” Total scores ranged from 8 to 32, with higher scores indicating greater perceived economic hardship.

Self-esteem was measured using the Rosenberg Self-Esteem Scale (RSES), originally developed by Rosenberg (27) and validated in Korean populations by Lee et al. (28). The scale consists of 10 items, each rated on a 4-point rating scale (1 = strongly disagree, 4 = strongly agree). An example item is “I feel that I am a person of worth, at least on an equal basis with others.” Total scores ranged from 10 to 40, with higher scores indicating higher self-esteem. In this study, the internal consistency of the self-esteem scale was acceptable, with a Cronbach’s alpha of 0.756.

Disability acceptance refers to a psychological adaptation process in which individuals acknowledge disability-related limitations while reconstructing personal values and assets beyond physical impairment (9). Disability acceptance was assessed using a 12-item scale rated on a 4-point rating scale (1 = strongly disagree, 4 = strongly agree). An example item is “I do not suffer because of my disability.” Total scores range from 12 to 48, with higher scores indicating greater acceptance of disability. In this study, the disability acceptance scale demonstrated acceptable internal consistency, with a Cronbach’s *α* of 0.789.

#### 2.3.2 Environmental factors

Environmental factors reflected social relationships, including the number of close persons and the frequency of contact with close persons. The number of close persons was assessed using the question, “How many people (e.g., friends or neighbors) do you have with whom you can discuss personal concerns? (Online contacts are excluded).” The frequency of contact with close persons was recategorized into two groups. Responses indicating contact almost daily, once or twice a week, or once or twice a month were classified as frequent contact, whereas responses indicating contact once or twice every 3 months, once or twice a year, or no contact were classified as rare contact.

#### 2.3.3 Health-related characteristics

Health-related characteristics included subjective health status, primary disability type, and disability severity, reflecting health-related and functional characteristics relevant to individuals’ disability status. Subjective health status was assessed using the question: “How would you rate your overall health during the past six months?” Responses of “very poor” and “poor” were categorized as poor, while “good” and “very good” were categorized as good. Primary disability type was classified as physical disability, brain lesion disability, visual impairment, hearing/speech impairment, intellectual or autism spectrum disorder, mental disorder, or internal/facial disability. Disability severity was categorized as mild or severe.

#### 2.3.4 Activities and participation

Activities were operationalized based on whether participants’ needs for assistance with activities of daily living (ADL/IADL) were met. This approach was adopted because the study population consisted of older adults with disabilities, for whom the ability to perform daily activities may depend not only on individual functional capacity but also on the availability of necessary assistance and support. Assistance covered domains such as personal hygiene, mobility, eating, dressing, health management, and participation in social and leisure activities. The level of assistance needed was assessed using a 4-point rating scale (1 = no assistance needed, 4 = complete assistance needed). The level of assistance received was measured on a parallel 4-point rating scale (1 = no assistance received, 4 = complete assistance received). Unmet needs for assistance were calculated as the difference between needed assistance and received assistance (needed − received). Values ≤ 0 were classified as needs met, whereas values > 0 were classified as unmet needs.

Participation was assessed through engagement in social activities, online social interaction, and leisure activities. Social activity participation was defined as engagement in religious activities, social gatherings, volunteering, or political/organizational activities at least once per month. Participation in at least one activity was coded as yes. Online social interaction was assessed based on the use of platforms such as Facebook, KakaoStory, Twitter, or Instagram. Participants using at least one platform were classified as yes. Leisure activity participation included activities such as watching television, listening to the radio, going to the movies, or exercising at least once per month. Participation in any activity was coded as yes.

#### 2.3.5 Life satisfaction

Life satisfaction, the dependent variable, was derived from the survey instrument used in the dataset and measured using eight items across multiple domains, including income, housing, education, employment, health, marital life, social relationships, and overall life satisfaction. Each item asked respondents to rate their level of satisfaction (e.g., “How satisfied are you with your current income?”) on a 10-point rating scale (1 = very dissatisfied, 10 = very satisfied). Total scores ranged from 8 to 80, with higher scores indicating greater life satisfaction. In this study, the internal consistency of the life satisfaction scale was acceptable, with a Cronbach’s alpha of 0.818.

### 2.4 Statistical analysis

Data analyses were conducted using SPSS. Descriptive statistics (frequencies, percentages, means, and standard deviations) were used to summarize participant characteristics and study variables. Differences in life satisfaction according to categorical study variables were examined using independent t-tests and one-way analysis of variance (ANOVA), with Scheffé *post hoc* tests applied when appropriate. Homogeneity of variance was examined using Levene’s test prior to independent t-tests. Pearson’s correlation coefficients were calculated to examine associations between life satisfaction and continuous study variables. To identify factors associated with life satisfaction, hierarchical multiple regression analysis was conducted based on the ICF-informed analytic framework.

## 3 Results

Figure 1 presents the analytic framework guiding the examination of associations among personal, environmental, health-related characteristics, and activities and participation in relation to life satisfaction.

### 3.1 Characteristics of the study variables

A total of 536 participants were included in the analysis. Regarding educational attainment, the largest proportion had completed elementary school (29.7%). The majority of participants (80.2%) were not employed. The mean scores for subjective economic hardship, self-esteem and disability acceptance were 17.18, 26.08 and 21.65, respectively. Participants reported an average of 1.73 close persons, and 45.5% indicated rare contact with close persons. More than half perceived their health status as poor (61.0%), and 39.9% were classified as having severe disabilities. Regarding activities and participation, 59.7% reported that their assistance needs for ADL/IADL were met, whereas engagement in online social interaction was notably low (3.5%). Detailed characteristics of the study variables are presented in Table 1.

**Table 1:** General characteristics of participants (*N* = 536).

Variables	Categories	*n*(%) /M ± SD
Personal factors		
Sex	Male	278 (51.9)
	Female	258 (48.1)
Education level	No formal education	62 (11.6)
	Elementary school	159 (29.7)
	Middle school	125 (23.3)
	High school	153 (28.5)
	College or higher	37 (6.9)
Employment status	Employed	106 (19.8)
	Unemployed	430 (80.2)
Subjective economic hardship		17.18 ± 4.83
Self-esteem		26.08 ± 4.87
Disability acceptance		21.65 ± 5.94
Environmental factor		
Number of close persons		1.73 ± 1.41
Frequency of contact with close persons	Frequent	292 (54.5)
	Rare	244 (45.5)
Health-related characteristics		
Subjective health status	Good	209 (39.0)
	Poor	327 (61.0)
Primary disability type	Physical disability	93 (17.4)
	Brain lesion disability	75 (14.0)
	Visual disability	93 (17.4)
	Hearing/speech disability	123 (22.9)
	Intellectual/autism disability	7(1.3)
	Mental disability	21(3.9)
	Internal/facial disability	124 (23.1)
Disability severity	Mild	322 (60.1)
	Severe	214 (39.9)
Activities and participation		
ADL/IADL assistance needs met	Met	320 (59.7)
	Unmet	216 (40.3)
Social activity participation	Yes	167 (31.2)
	No	369 (68.8)
Online social interaction	Yes	19 (3.5)
	No	517 (96.5)
Leisure activity participation	Yes	497 (92.7)
	No	39 (7.3)

Values are presented as number (percentage) or mean ± standard deviation.

ADL, activities of daily living; IADL, instrumental activities of daily living.

### 3.2 Differences in life satisfaction according to study variables

Significant differences in life satisfaction were observed across several personal, environmental, health-related, and activities and participation variables (Table 2). Specifically, female participants (*t* = −2.50, *p* = 0.013), participants who were employed (*t* = 12.76, *p* < 0.001), had frequent contact with close persons (*t* = 6.80, *p* < 0.001), perceived their health status as good (*t* = 11.92, *p* < 0.001), and had mild disabilities (*t* = −3.60, *p* < 0.001) reported significantly higher life satisfaction. In contrast, participants with brain lesion disabilities (*F* = 4.28, *p* < 0.001), unmet needs for assistance with daily activities (*t* = 5.11, *p* < 0.001), and no participation in social activities (*t* = 3.65, *p* < 0.001), and no participation in leisure activities (*t* = 2.30, *p* = 0.016) showed significantly lower life satisfaction.

**Table 2:** Differences in life satisfaction according to study variables (*N* = 536).

Variables	Categories	M ± SD	*t*/*F* (*p*)
Personal factors			
Sex	Male	24.09 ± 9.69	−2.50 (0.013)
	Female	26.21 ± 9.88	
Education level	No formal education	24.29 ± 8.87	0.50 (0.735)
	Elementary school	24.84 ± 10.02	
	Middle school	25.22 ± 10.13	
	High school	25.90 ± 9.68	
	College or higher	24.03 ± 10.37	
Employment status	Employed	35.58 ± 9.75	12.76 (<0.001)
	Unemployed	22.53 ± 7.97	
Subjective economic hardship		17.18 ± 4.83	
Environmental factor			
Frequency of contact with close persons	Frequent	27.59 ± 10.37	6.80 (<0.001)
	Rare	22.14 ± 8.22	
Health-related characteristics			
Subjective health status	Good	31.06 ± 10.23	11.92 (<0.001)
	Poor	21.31 ± 7.39	
Primary disability type	Physical disability^a^	27.71 ± 11.26	4.28 (<0.001) b < a, d
	Brain lesion disability^b^	21.17 ± 8.25	
	Visual disability^c^	24.87 ± 8.81	
	Hearing/Speech disability^d^	27.01 ± 10.74	
	Intellectual/Autism disability^e^	24.29 ± 7.06	
	Mental disability^f^	24.81 ± 7.49	
	Internal/Facial disability^g^	23.94 ± 8.99	
Disability severity	Mild	26.31 ± 10.24	−3.60 (<0.001)
	Severe	23.31 ± 8.89	
Activities and participation			
ADL/IADL assistance needs met	Met	26.85 ± 9.73	5.11 (<0.001)
	Unmet	22.53 ± 9.42	
Social activity participation	Yes	27.39 ± 9.41	3.65 (<0.001)
	No	24.08 ± 9.86	
Online social interaction	Yes	28.16 ± 12.04	1.38 (0.169)
	No	25.00 ± 9.74	
Leisure activity participation	Yes	25.38 ± 9.86	2.30 (0.016)
	No	21.64 ± 8.86	

ADL, activities of daily living; IADL, instrumental activities of daily living.

Superscripts a–g represent the primary disability types: a = physical disability; b = brain lesion disability; c = visual disability; d = hearing/speech disability; e = intellectual/autism disability; f = mental disability; g = internal/facial disability.

### 3.3 Correlations among main variables

Prior to conducting Pearson’s correlation analysis, assumptions of linearity and the absence of extreme outliers were examined and found to be satisfied. Correlation strength was interpreted based on conventional criteria, with coefficients of approximately 0.10 considered small, 0.30 moderate, and 0.50 large (29, 30). Pearson’s correlation analysis showed that life satisfaction was moderately positively correlated with self-esteem (*r* = 0.432, *p* < 0.001) and disability acceptance (*r* = 0.404, *p* < 0.001), and weakly positively correlated with the number of close persons (*r* = 0.298, *p* < 0.001). In contrast, subjective economic hardship was moderately negatively correlated with life satisfaction (*r* = −0.429, *p* < 0.001) (Table 3).

**Table 3:** Correlations among study variables (*N* = 536).

Variables	1	2	3	4	5
1. Subjective economic hardship	1				
2. Disability acceptance	−0.233 (<0.001)	1			
3. Self-esteem	−0.353 (<0.001)	0.498 (<0.001)	1		
4. Number of close persons	−0.224 (<0.001)	0.203 (<0.001)	0.254 (<0.001)	1	
5. Life satisfaction	−0.429 (<0.001)	0.404 (<0.001)	0.432 (<0.001)	0.298 (<0.001)	1

### 3.4 Factors associated with life satisfaction

Hierarchical multiple regression analysis was conducted using the ICF-informed analytic framework, with variables entered sequentially across four models. Personal factors were entered in Model 1, followed by environmental factors in Model 2, health-related characteristics in Model 3, and activities and participation factors in Model 4.

Model 1 explained 42.8% of the variance in life satisfaction (*R*^2^ = 0.428, *F* = 79.31, *p* < 0.001). The addition of environmental factors in Model 2 significantly increased the explained variance to 46.2% (Δ*R*^2^ = 0.034, *p* < 0.001). When health-related characteristics were included in Model 3, the explanatory power further increased to 51.8% (Δ*R*^2^ = 0.056, *p* < 0.001). The final model (Model 4), which included activities and participation factors, explained 52.9% of the variance in life satisfaction (*R*^2^ = 0.529, adjusted *R*^2^ = 0.518, *F* = 49.00, *p* < 0.001).

Within the personal factors domain, subjective economic hardship was negatively associated with life satisfaction (*β* = −0.151, *p* < 0.001), whereas employment status (*β* = 0.326, *p* < 0.001), self-esteem (*β* = 0.114, *p* = 0.002), and disability acceptance (*β* = 0.101, *p* = 0.006) were positively associated with life satisfaction. Among environmental factors, rare contact with close persons was negatively associated with life satisfaction (*β* = −0.121, *p* = 0.001). Regarding health-related characteristics, subjective health status was positively associated with life satisfaction (*β* = 0.256, *p* < 0.001). Within the activities and participation domain, unmet needs for assistance with activities of daily living (*β* = −0.078, *p* = 0.013) and non-participation in leisure activities (*β* = −0.073, *p* = 0.016) were negatively associated with life satisfaction (Table 4).

**Table 4:** Hierarchical multiple regression analysis predicting life satisfaction (*N* = 536).

Predictor	Model 1	Model 2	Model 3	Model 4
	*β* (*p*)	*β* (*p*)	*β* (*p*)	*β* (*p*)
Sex (female)	0.041 (0.214)	0.034 (0.287)	0.029 (0.342)	0.027 (0.381)
Employment status (employed)	0.361 (<0.001)	0.347 (<0.001)	0.334 (<0.001)	0.326 (<0.001)
Subjective economic hardship	−0.182 (<0.001)	−0.171 (<0.001)	−0.159 (<0.001)	−0.151 (<0.001)
Self-esteem	0.146 (<0.001)	0.136 (<0.001)	0.121 (0.004)	0.114 (0.002)
Disability acceptance	0.132 (<0.001)	0.121 (0.002)	0.109 (0.004)	0.101 (0.006)
Number of close persons	—	0.062 (0.114)	0.053 (0.168)	0.048 (0.199)
Contact with close persons (rare)	—	−0.149 (<0.001)	−0.132 (0.002)	−0.121 (0.001)
Disability severity (mild)	—	—	0.028 (0.371)	0.021 (0.495)
Subjective health status (good)	—	—	0.279 (<0.001)	0.256 (<0.001)
ADL/IADL assistance needs met (unmet)	—	—	—	−0.078 (0.013)
Social activity participation (no)	—	—	—	0.001 (0.969)
Leisure activity participation (no)	—	—	—	−0.073 (0.016)
*R* ^2^	0.428	0.462	0.518	0.529
Adjusted *R*^2^	0.423	0.455	0.510	0.518
Δ*R*^2^	0.428	0.034	0.056	0.011
*F* (*p*)	79.31 (<0.001)	64.70 (<0.001)	62.79 (<0.001)	49.00 (<0.001)

Standardized regression coefficients (*β*) with corresponding *p*-values are reported.

Reference categories: male, unemployed, severe disability, poor subjective health, frequent contact, needs met, participation yes.

## 4 Discussion

This study applied an ICF-informed analytic framework to examine factors associated with life satisfaction among older adults with disabilities living alone. As illustrated in Figure 1, life satisfaction was conceptualized as an outcome associated with interrelated personal, environmental, health-related characteristics, and activities and participation factors. The findings demonstrated that life satisfaction was significantly associated with variables across multiple domains, including personal factors (subjective economic hardship, employment status, self-esteem, and disability acceptance), environmental factors (frequency of contact with close persons), health-related characteristics (subjective health status), activities and participation factors (whether needs for assistance with activities of daily living were met and leisure activity participation).

Taken together, these findings highlight the multidimensional nature of life satisfaction among older adults with disabilities and support the usefulness of an ICF-informed framework for organizing and interpreting multidimensional associations related to life satisfaction.

Among personal factors, disability acceptance was found to have a significant association with life satisfaction. Disability acceptance is a psychological adaptation process associated with lower negative emotional responses to disability and greater perceived life meaning. It has been conceptualized as a stress-buffering mechanism that directly enhances well-being and life satisfaction (11, 12). The present findings suggest that interventions aimed at enhancing disability acceptance—such as group counseling, peer support, and meaning-centered interventions—may be beneficial for improving life satisfaction among older adults with disabilities. Self-esteem was also identified as a significant predictor of life satisfaction, consistent with prior research demonstrating that self-esteem plays a central role in subjective well-being (31, 32). As a core internal resource, self-esteem enhances perceived control, positive self-evaluation, and resilience in the face of stress, and has been consistently associated with higher life satisfaction (32). These findings support the need for strength-based approaches that recognize older adults with disabilities not merely as recipients of support, but as individuals with capabilities and agency. Programs in vocational rehabilitation, lifelong learning, leisure, and social participation that provide opportunities for mastery experiences and social recognition may be particularly effective.

Regarding environmental factors, the number of close persons was not significantly associated with life satisfaction in the final model, whereas the frequency of contact with close persons remained significant. This finding is consistent with previous research suggesting that the size of one’s social network does not necessarily translate into higher life satisfaction (15). Berkman et al. (15) emphasized that the functional aspects of social relationships—how relationships are activated and experienced—are more important than the sheer number of social ties. For older adults with disabilities, who often face mobility limitations, environmental barriers, and social stigma, the presence of relationships alone may be insufficient; rather, actively maintained and emotionally meaningful relationships may exert a more direct influence on life satisfaction (17). The significance of contact frequency suggests that life satisfaction is shaped through repeated emotional exchanges and sustained interactions (33). Holt-Lunstad et al. (16) conceptualized social connectedness as a combination of ongoing contact, emotional closeness, and a sense of social integration. From this perspective, frequency of contact may serve as a proxy for experienced emotional support, belongingness, and social presence, thereby exerting a more immediate influence on life satisfaction than network size.

With respect to health-related characteristics, better subjective health status was significantly associated with higher life satisfaction. This finding is consistent with previous research among people with disabilities in Taiwan, which reported that individuals who perceived their health more positively had significantly higher levels of life satisfaction (17). Similarly, Bramhankar et al. (18) reported that older adults who rated their health as “good” were approximately five times more likely to report higher life satisfaction compared to those who rated their health as “poor,” highlighting self-rated health as a key predictor of life satisfaction. These findings suggest that when individuals perceive their health status positively, they are more likely to maintain functional capacity in daily life, which has been shown to be associated with higher life satisfaction. Moreover, subjective health perception has been reported as a factor that can mitigate activity limitations and facilitate connections to social participation among people with disabilities (34). Therefore, improvements in functional status should not be understood solely as the alleviation of disease or physical impairment, but rather as a foundational condition that enables activity and participation.

In the activities and participation domain, whether needs for assistance with activities of daily living (ADL/IADL) were met was significantly associated with life satisfaction. This finding aligns with previous studies showing that the timely and appropriate provision of assistance for ADL and IADL needs is closely associated with higher life satisfaction (19). Within the ICF framework, activity encompasses not only an individual’s ability to perform tasks but also the contextual conditions under which those tasks are carried out (25). Meeting assistance needs at the activity level may be related to reduced perceived functional limitations and greater participation. When necessary assistance is not adequately provided, individuals may experience reduced functional autonomy and perceived control, leading to lower life satisfaction. Conversely, when support at the activity level is well aligned with individual needs, subjective health perception and self-efficacy are more likely to be maintained or strengthened, ultimately contributing to greater life satisfaction (17, 18). These findings underscore the importance of enhancing the appropriateness of activity-level support for older adults with disabilities.

Participation in leisure activities emerged as a significant factor associated with higher life satisfaction. Leisure participation has been widely recognized as a key contributor to autonomy and psychological well-being among older adults with disabilities (35, 36). Prior studies suggest that leisure activities that individuals plan and choose themselves enhance self-determination and perceived control by allowing them to reflect their interests and preferences, thereby fostering positive emotions, life satisfaction, and self-esteem (35). In addition, leisure, cultural, and physical activities function not merely as means of passing time, but as important contexts for social interaction and role performance, contributing to reduced social isolation and expanded social networks (36, 37). Through these processes, leisure participation is associated with partial compensation for physical functional limitations and with the reconstruction of life meaning and social roles following disability (38). Accordingly, leisure participation should be understood not simply as an indicator of activity frequency, but as a marker of whether older adults with disabilities are able to actively reconstruct meaning in daily life and function as social members. These findings highlight the need for environmental supports, such as accessible leisure facilities and tailored programs, to promote participation among older adults with disabilities.

Furthermore, the findings of this study should be interpreted within the specific sociocultural and policy context of South Korea. The living conditions and life satisfaction of older adults with disabilities are shaped by country-specific welfare systems, disability policies, family norms, and social expectations regarding aging and care. In South Korea, relatively limited community-based support and a strong reliance on family or informal networks may influence both social participation patterns and perceived life satisfaction among older adults with disabilities living alone (39). Therefore, caution is warranted in generalizing the present findings to other countries with different welfare regimes and social care infrastructures. Comparative studies across diverse policy and cultural contexts are needed to further examine the applicability of these findings.

Overall, the findings of this study indicate that life satisfaction among older adults with disabilities living alone is shaped by the interaction of personal, psychological, social, and environmental factors. Strengthening the virtuous cycle in which functional capacity enables activity and activity expands participation requires simultaneous interventions targeting both individual-level resources and environmental supports. Accordingly, rehabilitation and social welfare policies for older adults with disabilities should move beyond a narrow focus on physical function recovery and adopt an integrated approach that supports expanded activity opportunities, enhanced social participation, and psychosocial adaptation.

This study has several limitations. First, the use of secondary data and a cross-sectional design limits the ability to establish causal relationships among variables. Second, the age criterion of 60 years or older may limit direct comparisons with studies defining older adulthood as 65 years or older. In addition, the findings should be interpreted within the analytic scope of the study. Although the ICF framework emphasizes interactions between individuals and broader environmental contexts, including community, institutional, and policy-level factors, the present analysis was limited to immediate relational and individual-level environmental variables due to the constraints of the secondary data. Consequently, broader structural conditions shaping opportunities for social participation and sustained social contact could not be fully examined, which may limit the explanatory scope of the findings.

Several measurement-related limitations should also be considered. Some variables, including subjective health status, social contact, and participation, were dichotomized due to the distributional characteristics of the secondary data, which may have reduced variability and explanatory power. In particular, the high prevalence of leisure activity participation suggests a potential ceiling effect that may have attenuated observed associations with life satisfaction. Furthermore, the activity-related variable reflected whether assistance needs for ADL/IADL were met, which may capture both activity limitations and the availability of environmental support rather than activity limitation alone as strictly defined within the ICF framework. Future research should employ longitudinal designs, incorporate broader environmental and policy-level variables, and utilize more granular measures of activity and participation within ICF-informed analytic frameworks.

In conclusion, this study applied an ICF-informed analytic framework to examine factors associated with life satisfaction among older adults with disabilities living alone. The findings demonstrated that life satisfaction was associated with multidimensional factors across personal, environmental, health-related, and activities and participation domains. Specifically, subjective economic hardship, unmet needs for assistance with activities of daily living, non-participation in leisure activities, and infrequent contact with close persons were negatively associated with life satisfaction, whereas employment status, disability acceptance, self-esteem, and subjective health status were positively associated.

These findings highlight the importance of considering rehabilitation and social welfare policies for older adults with disabilities living alone that extend beyond a sole focus on physical function recovery. Instead, integrated strategies are needed that ensure the appropriateness of support at the level of activities of daily living, expand opportunities for leisure and social participation, foster environments that emphasize the quality rather than the quantity of social relationships, and incorporate psychosocial interventions aimed at enhancing disability acceptance and self-esteem. In particular, the identification of unmet needs for assistance with activities of daily living and the frequency of social contact as key predictors of life satisfaction highlights the need for policy designs that prioritize functioning support and social connectedness that actively operate in daily life, rather than merely increasing potential resources.

## Data Availability

Publicly available datasets were analyzed in this study. This data can be found here: The data were obtained from the fourth wave of the Disability and Life Dynamics Panel (DLDP), conducted by KODDI, and are available through the Disability Statistics Data Portal (https://koddi.or.kr/stat/html/user/main/main) upon application and approval.
